# The Association Between Hypothyroidism and Vitamin D Deficiency in Non-Alcoholic Fatty Liver Disease in Overweight or Obese Female Adults: A Case-Control Study

**DOI:** 10.34172/hpp.44592

**Published:** 2026-08-21

**Authors:** Nazanin Pourseyedi, Sara Arefhosseini, Helda Tutunchi, Saeed Ostadrahimi, Mehrangiz Ebrahimi-Mameghani

**Affiliations:** ^1^Student Research Committee, Tabriz University of Medical Sciences, Tabriz, Iran; ^2^Endocrine Research Center, Tabriz University of Medical Sciences, Tabriz, Iran; ^3^Department of Chemical and Biochemical Engineering, University of Western Ontario, London, ONN6A 5B9, Canada; ^4^Nutrition Research Center, Department of Biochemistry and Diet Therapy, Faculty of Nutrition and Food Sciences, Tabriz University of Medical Sciences, Tabriz, Iran

**Keywords:** Hypothyroidism, Non-alcoholic fatty liver disease, Obesity, Vitamin D

## Abstract

**Introduction:**

While evidence suggests a link between serum vitamin D levels and hypothyroidism, this relationship remains unclear, particularly within non-autoimmune hypothyroid conditions. This study aimed to investigate the association between vitamin D deficiency (VDD) and non-autoimmune hypothyroidism in overweight/obese patients with non-alcoholic fatty liver disease (NAFLD).

**Methods:**

This case-control study included 154 overweight or obese (body mass index [BMI]≥25 kg/m^2^) female adults diagnosed with NAFLD based on ultrasonography findings. Seventy-seven patients with NAFLD and VDD (serum 25(OH)D concentration≤30 nmol/L) as the "*Case*" group and Seventy-seven age, and BMI-matched patients with NAFLD without VDD as the "*Control*" group were enrolled. Anthropometric parameters and serum levels of alanine aminotransferase (ALT), aspartate aminotransferase (AST), free triiodothyronine (FT3), free thyroxine (FT4) and thyroid stimulating hormone (TSH) were measured.

**Results:**

The baseline characteristics of the study showed no significant differences in age, anthropometric measures, or physical activity levels between the two groups. Analysis of weekly frequency consumption of vitamin D-rich foods revealed no notable differences between VDD cases and controls, except for a statistically significant difference in the consumption of low-fat cheese (*P*=0.024). Additionally, no significant association was identified between TSH levels and VDD in either crude or adjusted models, after accounting for potential confounders, including sun exposure duration, BMI, and serum ALT and AST levels. However, participants with low sun exposure (≤1 hour/day) were significantly more likely to have VDD (OR=3.62; 95% CI=1.52-8.60).

**Conclusion:**

Our results did not show association between serum vitamin D levels and non-autoimmune hypothyroidism in patients with NAFLD.

## Introduction

 Non-alcoholic fatty liver disease (NAFLD) has become the most common cause of chronic liver disease globally, evolving from a relatively unknown disease.^1^ It covers a wide range, from simple hepatic steatosis to nonalcoholic steatohepatitis (NASH), which may lead to liver fibrosis and cirrhosis.^2^ The diagnostic criteria for NAFLD rely on ‘ruling out’ hepatic steatosis due to other factors such as alcohol, viral hepatitis, and drugs.^3^ The global prevalence of NAFLD continues to rise, currently affecting an estimated 32% of the adult population.^4^

 The pathogenesis of NAFLD involves multifactorial mechanisms, and the “multiple-hit hypothesis” offers a framework for understanding its development and progression.^5^ Genetic susceptibilities, dietary habits, lifestyle factors, metabolic dysfunction (primarily insulin resistance (IR)), and alterations in the gut microbiome all contribute to the development of more advanced liver disease either simultaneously or sequentially.^3^ In individuals with genetic predisposition or epigenetic modifications, these factors influence the fat content of hepatocytes and the inflammatory environment, leading to chronic hepatic inflammation.^6^ IR plays a critical role in the development of steatosis/NASH as the “first hit” which leads to increased hepatic de novo lipogenesis (DNL) and impaired inhibition of adipose tissue lipolysis, and finally, an increased flow of fatty acids to the liver.^7^ Furthermore, mitochondrial dysfunction and oxidative stress contribute to lipotoxicity, further exacerbating liver damage.^8,9^ NAFLD is closely associated with IR, obesity, and type 2 diabetes mellitus (T2DM), which result in the development of adverse hepatic and extra-hepatic outcomes.^10^ Emerging evidence suggests that vitamin D may play a role in these metabolic processes, as it has critical functions in IR, inflammation modulation, and adipose tissue (AT) regulation.^11^ Given that AT serves as a primary storage site for vitamin D and vitamin D receptors (VDR) are present in the liver, vitamin D deficiency (VDD) may exacerbate metabolic dysfunctions that drive NAFLD progression.^12,13^ Numerus studies suggest a link between obesity and VDD.^11,14-16^ The potential role of VDD in obesity development has been investigated, as AT is a direct target for the effects of vitamin D.^17^ The expression of both VDR and the 25-hydroxyvitamin D 1α-hydroxylase genes in adipocytes indicates that these cells may participate in the local production of biologically active 1,25(OH)2D, which could then bind to the VDR, exert endocrine, autocrine, or paracrine effects on AT.^18^ Due to its critical involvement in metabolic processes, VDD has been recognized as a major contributing factor in the development and progression of NAFLD as well as its associated complications,^12^ as well as one of the most common global health issues, affecting individuals across all age groups, including those in low-latitude regions with abundant UV exposure and industrialized countries with long-standing vitamin D fortification programs ^19^ such as Iran with 50% prevalence rate.^20^ This widespread deficiency is concerning due to its associations with various metabolic and autoimmune diseases, including thyroid disorders.^21^ On the other hand, thyroid hormones (THs) play a powerful role in regulating cellular development, growth, and metabolism that regulate various metabolic processes and maintain stability in different physiological conditions.^22^ In addition to its critical regulatory role in cellular homeostasis, the imbalance of THs levels is associated with multiple chronic diseases, including diabetes mellitus, cardiovascular disease, and liver-related disorders.^23,24^ The liver is a central organ for TH metabolism, influencing beta-oxidation, cholesterol, and carbohydrate regulation.^25^ Hypothyroidism, therefore, is often associated with dyslipidemia, including elevated low-density lipoprotein cholesterol (LDL-C) and total cholesterol levels.^26^ There is evidence indicating a potential link between hypothyroidism and VDD, likely due to shared nuclear hormone receptors involved in both vitamin D and TH activity.^27,28^ While numerous studies have investigated the association between VDD and hypothyroidism, most of these have focused exclusively on autoimmune hypothyroidism named as Hashimoto disease.^29^ This focus has constrained a comprehensive understanding of the potential role of vitamin D in non-autoimmune hypothyroidism, where underlying mechanisms and associations may differ from those in autoimmune forms.^30,31^ Therefore, this case-control study was designed to investigate the relationship between VDD and non-autoimmune hypothyroidism in patients with NAFLD, thereby offering insight into a less-explored hypothyroid population.

## Methods

###  Study design 

 The present case-control study was conducted on 154 female adults, aged 18-50 years, body mass index (BMI) ≥ 25 kg/m^2^ and NAFLD confirmed by ultrasonography findings in Tabriz, Iran between September 2023 and March 2024. Seventy-seven patients with VDD (serum 25-hydroxyvitamin D concentration ≤ 30 ng/mL) were enrolled as “cases”, and seventy-seven subjects without VDD were enrolled as “controls”, with frequency matching based on age group and BMI status.^32^ The specialist rulled out autoimmune hypothyroidism or Hashimoto disease based on Anti-TPO test (according to the manufacturer’s recommended cut-off value, i.e. > 30 IU/mL) and referred those with hypothyroidism to the researcher.

 The subjects who were pregnant, lactating, menopause, athlete, using alcohol or cigarette, bariatric surgery, suffering from other chronic liver disease, liver transplantation recipients, gallbaldder disease, autoimmune disorders, polycystic ovary syndrome, malabsorption diseases, cancers, thyroid medications, including levothyroxine and antithyroid drugs, chemical or herbal anti-obesity medications, hepatotoxic drugs, anti-hypertensive drugs, taking lipid- or glucose- lowering medications, antacids, oral contraceptives and estrogen, corticosteroids drugs, nonsteroidal anti-inflammatory drugs, strict diets, vitamin-mineral supplements over last three months were excluded. The study protocol was approved by the Ethics Committee of the Tabriz University of Medical Science (IR.TBZMED.REC.1402.617), and all participants signed an informed written consent form.

####  Sample size 

 The sample size was calculated based on the results reported by Jablonski et al. (OR = 2.4) ^33^ a confidence interval (CI) of 95% (2-tailed), 80% power, the proportion of exposure in paatients (PO = 54%) and in controls (p1~ 76%), allocation ratio 1:1. The sample size was estimated at 77 subjects for each group.

####  Data collection

 All participants were individually interviewed to collect data on demographics, socioeconomic status, and health-related behaviors including smoking, alcohol consumption, medical history, and supplement intake by a trained nutritionist. Weight and height were measured using a calibrated stadiometer (Seca, Germany) with light clothing and no shoes to the nearest 0.1 kg and 0.1 cm, respectively. Waist circumference (WC) and hip circumference (HC) were measured using non-stretchable tape at the midpoint between the lower border of the rib cage and iliac crest and around the widest part of the buttocks to the nearest 0.5 cm, respectively. BMI, waist-to-hip ratio (WHR), and waist-to-height ratio (WHtR) were calculated as obesity indices. Moreover, the participants were asked about their average daily duration and timing of sunlight exposure. Total daily sun exposure was estimated by the sum of sun exposure at morning, noon and afternoon and then, it was used as possible confounder for VDD.

####  Laboratory assessment 

 Blood sample (5 ml) was collected after a 12-hour overnight fast from each participant and then centrifuged. The collected serum was stored at -20°C until the end of the study. Serum alanine aminotransferase (ALT) and aspartate aminotransferase (AST) levels were determined using the Pars Azmoon kit and an auto-analyzer (Hitachi 902, Tokyo, Japan), following the International Federation of Clinical Chemistry and Laboratory Medicine (IFCC) procedure.^34^ The serum concentration of 25(OH)D was measured using high-performance liquid chromatography (HPLC) (series 1100, Germany).^35^ The concentration of T3, T4 and TSH were determined using the Electrochemiluminescence (ECL) method and the enzyme-linked immunosorbent assay (ELISA) kits.^36^

####  Physical activity assessment 

 To control the confounding effects of physical activity, a validated International Physical Activity Questionnaire Short Form (IPAQ-SF) was completed by the patients. The questionnaire assessed participants’ physical activity over the previous week through seven questions on activity type and duration. Participants reported time spent on vigorous activities (e.g., heavy lifting, fast cycling), moderate activities (e.g., carrying light loads, regular-paced cycling), and walking (for work, home, or leisure). Total metabolic equivalent task (MET-minutes per week) score were calculated for the participants using an Excel program and in accordance with the manual. Average METs of 3.3, 4.0, and 8.0 were used for walking, moderate, and vigorous activities, respectively. These values were multiplied by the minutes spent on each activity and summed to calculate the overall METs-minute score for the week. The participants were divided into three categories based on their level of activity: high (≥ 3000 MET-minutes/week), moderate (600-3000 MET-minutes/week), or low (< 600 MET-minutes/week).^37^

###  Statistical analysis

 Data was analyzed using (IBM SPSS Statistics, version 20; IBM Corp, Armonk, LA, USA). To check the normality distribution of continuous variables, Kolmogorov-Smirnov test was applied. Continuous data were presented as mean ± standard deviation [SD]) or median and interquartile range (25^th^, 75^th^) and categorical data were reported as number (%), respectively. Chi-square test was used for the relationship between categorical variables. Differences in symmetric and asymmetric continuous variables were done using two- independent sample t- and Mann-whitney U-tests. Association between continuous variables was also checked using Pearson and Spearman correlation coefficient for symmetric and asymmetric data, respectively. The relationship between VDD and hypothyroidism was tested using logistic regressionby considering VDD as dependent outcome. As sun exposure and serum levels of asminotransferases were found as confounders because of significant diffrences netween the groups aswell as BMI value to be conservative in the interpreteation (Table 1). Uni- and Multivariable logistic regression was performed to report odds ratios (OR) with 95% confidence interval (CI) were reported. *P* < 0.05 was considered as statistically significant.

**Table 1:** Baseline characteristics of participants

**Variables**	**VDD** **(N=77)**	**Non-VDD** **(N=77)**	***P*** value*
Age (years)	35.68 (9.09)	37.71 (9.16)	0.168^*^
Weight (kg)	86.67(11.06)	87.75 (10.30)	0.542^*^
Height (cm)	160.63 (7.49)	160.12 (6.78)	0.660^*^
WC (cm)	106.63 (8.80)	100.15 (8.87)	0.835^*^
HC (cm)	118.02 (12.94)	116.79 (7.58)	0.475^*^
WHR	0.91 (0.07)	0.92 (0.07)	0.457^*^
WHtR	0.67 (0.06)	0.67 (0.06)	0.724^*^
BMI (Kg/m^2^)	33.53 (2.96)	34.21 (3.38)	0.180^*^
FBS (mg/dL)	97.40 (21.07)	93.41 (14.29)	0.136^*^
HbA1c (%)	5.50 (5.20, 5.65)	5.40 (5.20, 5.50)	0.252^*^
TG (mg/dL)	164.62 (9.03)	144.96 (8.34)	0.122^*^
TC (mg/dL)	186.41 (45.82)	186.49 (42.88)	0.990^*^
Uric acid (mg/dL)	5.07 (1.32)	4.96 (1.39)	0.607^*^
ALT (IU/L)	24.0 (19.5, 34.0)	21.0 (16.0, 29.25)	0.043^**^
AST (IU/L)	22.0 (18.20, 31.0)	19.0 (15.5, 25.5)	0.009^**^
Ferritin (ng/mL)	57.90 (40.30, 77.45)	48.5 (28.65, 69.80)	0.021^**^
T3 (nmol/L)	157.0 (117.5, 159.0)	140.0 (120.0, 170.0)	0.222^**^
T4 (nmol/L)	6.78 (3.11, 7.98)	5.57 (2.26, 7.45)	0.210^**^
TSH(μIU/mL)	2.10 (1.37, 3.21)	1.80 (1.46, 3.16)	0.487^**^
25(OH)D (ng/mL)	21.40 (17.25, 24.80)	44.0 (36.0, 53.0)	< 0.001^**^
Calcium (mg/dL)	9.60 (0.34)	9.58 (0.52)	0.255^*^
Phosphourus (mg/dL)	3.65 (0.43)	3.41 (0.51)	0.993^*^
Parts of body exposed to sunlight			0.163^***^
Face	17 (22.0)	0	
Wrist	0 (0)	7.1	
Arm and forearm	3 (3.9)	7.1	
Legs	7 (9.1)	0	
Face and wrist	50 (65.0)	85.8	
Sunscreen status			0.130^***^
Never	23 (29.9)	11 (14.3)	
Sometimes	10 (13.0)	22 (28.6)	
Often	24 (31.2)	5 (6.5)	
Always	20 (25.9)	39 (50.6)	
Sun exposure frequency			0.355^***^
Sometimes	10 (13.0)	0 (0)	
Rarely	6 (7.8)	5 (6.5)	
Never	61 (79.2)	72 (93.5)	
Duration of sun exposure (min/day)			0.034
Morning	28.33 (21.30)	44.84 (34.29)	
Noon	34.79 (21.96)	40.54 (38.57)	
Afternoon	22.14 (7.83)	11.33 (6.43)	
Total sun exposure time /day ≤ 1 hr 1 hr <	67 (87.0) 10 (13.0)	52 (67.5) 25 (32.50)	0.004*
Physical activity level (MET-min/wk) Low (< 600) Moderate (600-3000)	45 (58.44) 32 (41.56)	50 (64.93) 27 (35.07)	0.410^***^

WC, waist circumference; HC, hip circumference; WHR, waist to hip ratio; WHtR, waist to height ratio; BMI, body mass index; PA, physical activity; FBS, fasting blood sugar; HbA1c, hemoglobin A1c; TG, triglycerides; TC, total cholesterol; ALT, alanine transaminase; AST, aspartate transaminase; T3, triiodothyronine; T4, thyroxine; TSH, thyroid stimulating hormone ^*^ *P* for Independent sample t-test, ^**^P for Mann-Whitney U-test, ^***^P for Chi square test

## Results

 In this case-control study, 77 VDD participants and 77 age and BMI-matched non-VDD participants were included. Table 1. shows baseline characteristics of the participants. There were no significant differences in age, anthropometric measures as well as physical activity level between the groups. However, between group differences in serum levels of ALT, AST, and ferritin were statistically significant. Furthermore, no significant differences were found in thyroid related hormones. Regarding sunexposure, only in patients with VDD, total daily sun exposure time was significantly less than those without VDD (*P* = 0.004).

Table 2. shows that there were no significant differences in the frequency consumption of vitamin D rich foods between the VDD cases and controls, apart from low-fat cheese.

**Table 2:** Weekly frequency consumption of foods

**Variables**	**VDD** **(N=77)**	**Non-VDD** **(N=77)**	***P*** value*
Beef	2.00 (0.00,3.00)	1.00 (0.00,2.00)	0.353
Lamb	0.85 (0.00,3.00)	1.00 (0.00,2.50)	0.880
Minced meat	1.50 (0.865,3.00)	1.00 (1.00,2.50)	0.960
Liver	2.00 (0.00,0.11)	0.05 (0.00,0.21)	0.801
Chicken	2.50 (1.00,3.25)	3.00 (3.00,3.50)	0.216
Egg	2.50 (0.64,4.25)	3.00 (1.00,4.50)	0.987
Liver poultry	0.00 (0.00,0.00)	0.00 (0.00,0.05)	0.353
Fish	0.05 (0.025,0.23)	0.11 (0.005,0.23)	0.801
Fish tuna	0.07 (0.00,0.345)	0.11 (0.00,0.46)	0.880
Shrimp	0.00 (0.00,0.05)	0.00 (0.00,0.015)	0.724
Bologna	0.11 (0.01,0.23)	0.17 (0.022,0.70)	0.724
Sausage	0.09 (0.00,0.23)	0.07 (0.02,0.23)	0.775
Hamburger	0.23 (0.03,1.00)	0.23 (0.08,0.46)	0.724
Pizza	0.11 (0.025,0.46)	0.11 (0.05,0.23)	0.555
Mixed pizza	0.00 (0.00,0.07)	0.00 (0.00,0.17)	0.169
Rumen	0.00 (0.00,0.015)	0.00 (0.00,0.04)	0.489
Tongue	0.00 (0.00,0.01)	0.00 (0.00,0.05)	0.699
Brain	0.00 (0.00,0.02)	0.00 (0.00,0.00)	0.749
Head	0.00 (0.00,0.00)	0.00 (0.00,0.00)	0.649
Low fat milk	0.00 (0.00,2.00)	0.23 (0.00,3.50)	0.243
High fat milk	0.00 (0.00,0.70)	0.00 (0.00,0.20)	0.649
Chocolate milk	0.00 (0.00,1.00)	0.00 (0.00,0.23)	0.468
Low fat yogurt	0.00 (0.00,1.00)	0.23 (0.00,2.50)	0.271
High fat yogurt	0.055 (0.00,3.00)	0.19 (0.00,1.50)	0.906
Strained yogurt	0.00 (0.00,2.00)	0.00 (0.00,0.015)	0.191
Low fat cheese	0.00 (0.00,1.00)	2.00 (0.005,7.00)	**0.024**
Lighvan cheese	4.50 (1.75,7.00)	3.00 (0.00,7.00)	0.257
Cream cheese	0.00 (0.00,0.11)	0.00 (0.00,0.14)	0.749
Pasteurized buttermilk	0.00 (0.11,0.46)	0.23 (0.00,1.00)	0.933
Local buttermilk	0.00 (0.00,0.04)	0.00 (0.00,0.85)	0.353
Curd	0.06 (0.00,0.14)	0.11 (0.015,0.23)	0.353
Cream	0.03 (0.00,1.00)	0.09 (0.01,0.38)	0.625
Pasteurized ice cream	0.17 (0.00,0.852)	0.23 (0.08,0.78)	0.371
Ghee and butter	0.00 (0.00,0.595)	1.00 (0.00,3.00)	0.960
Pasteurized butter	0.025 (0.00,1.00)	0.23 (0.00,1.00)	0.724
Margarine	0.00 (0.00,0.00)	0.00 (0.00,0.00)	1.000
Fresh mushroom	0.23 (0.037,0.947)	0.46 (0.08,2.50)	0.428
Canned mushroom	0.00 (0.00,0.00)	0.00 (0.00,0.00)	0.827

VDD; Vitamin D deficient *Mann-Whitney U-test Data given as median (25^th^, 75^th^)

Table 3. reveals the association between VDD and hypothyroidism in the studied patients. Results of crude logistic regression showed significant association between totaldaily sun exposure time, serim ALT and AST with VDD in patients with NAFLD. However, after controlling for the latter variables as well as BMI, we found that there was no relationship between TSH level and VDD while patients with low sun exposure (≤ 1 hour/day) were 3.6 times more likely to suffer from VDD (OR = 3.61, CI 95% = 1.52,8.60) compared with those with sun exposure more than one hour/day.

**Table 3:** Association between VDD and hypothyroidism

	**Unadjusted OR (CI95%)**	***P*** *	**Adjusted OR** ** (CI 95%)**	***P*** **
TSH (μIU/mL)	0.999 (0.82,1.20)	0.924	0.999 (0.82,1.22)	0.995
Total Sun exposure/day		0.005		0.004
≤ 1 hour	3.22 (1.42,7.30)		3.61 (1.52,8.60)	
> 1 hour	1.00		1.00	
ALT (IU/L)	1.04 (1.01,1.07)	0.018	1.01 (0.96,1.07)	0.597
AST (IU/L)	1.05 (1.01,1.10)	0.011	1.04 (0.97,1.12)	0.227
BMI (Kg/m^2^)	0.93 (0.84,1.03)	0.180	0.95 (0.85,1.06)	0.331

VDD, vitamin D deficiency; TSH, thyroid stimulating hormone; ALT, alanine transaminase; AST, aspartate transaminase; BMI, body mass index * *P* for unadjusted logestic regression ** *P* for adjusted for serum TSH, total daily sun exposure/day, ALT, AST and BMI

## Discussion

 VDD is linked to various metabolic and endocrine disorders, and previous research has suggested potential link between low vitamin D levels and hypothyroid function.^28^ NAFLD- a common liver disease linked to metabolic syndrome- has been found to be associated with both VDD and hypothyroidism.^13,38^ The results of the present case-control study showed no significant link between hypothyroidism and serum vitamin D levels among overweight and obese female adults with NAFLD. Our study stands apart from most of the existing literature because this study focused exclusively on non-autoimmune hypothyroidism. The majority of studies on VDD and hypothyroidism have focused primarily on autoimmune hypothyroidism, particularly Hashimoto’s thyroiditis. This suggests that the link to vitamin D levels likely originates from immunological mechanisms.^39-41^

 Idiculla et al. in their cross-sectional study on 235 participants (115 subjects with primary hypothyroidism (including three cases of subclinical hypothyroidism) and 120 euthyroid controls). found that 93% of the participants were VDD. While the overall prevalence of VDD was not significantly different between the hypothyroid and euthyroid groups, severe VDD was higher among patients with hypothyroidism.^42^ Another cross-sectional study conducted by Saha et al. in 2023 on 100 previously diagnosed adult hypothyroid patients showed a positive correlation between T3 and T4 levels and vitamin D, while a negative correlation was found between vitamin D and serum TSH levels.^43^ As case-control design provides relatively stronger framework for exploring potential cause-and-effect relationships than cross-sectional design, this methodological difference could also account for the variations observed in the findings. A nested case-control carried out by Mirhosseini et al. on (103 hypothyroid patients in the case group and 412 healthy participants in control group) in 2017 ^44^ showed a link between hypovitaminosis D and thyroid autoimmunity. Similarly, Ahi et al. in a case-control study on 1,138 participants (633 autoimmune hypothyroid patients, 305 non-autoimmune hypothyroid patients, and 200 healthy individuals) found significantly lower vitamin D levels in both immune and non-immune Hashimoto’s thyroiditis compared to healthy controls (*P* < 0.05).^45^ However, a case-control study on 116 female participants in 2017 failed to find any significant difference in serum 25(OH)D between those with hypothyroidism and healthy controls.^46^ Shankar et al.conducted a cross-sectional study in 2020 on 90 women, found no correlation between serum vitamin D levels in hypothyroidism and healthy controls.^47^ Notably, the latter two studies that did not align with our findings included both genders, while our study exclusively focused on females. This difference in study populations could partly explain the variations in the studied outcomes, as gender-specific factors may influence the relationship between vitamin D levels and hypothyroidism. A meta-analysis study of 20 case-control studies conducted by Wang et al. on 994 autoimmune thyroid disease patients and 1035 healthy participants found lower levels of 25(OH)D in individuals with autoimmune thyroid disease than healthy individuals.^48^ In a systematic review and meta-analysis of observational studieson 42 studies (9 studies included hypothyroid patients, 13 studies included autoimmune thyroid disease patients, 12 studies included Hashimoto’s thyroiditis patients, and 8 studies included Grave’s disease patients) and all studies assessed the serum levels of 25(OH)D and none of them examined dietary intake on vitamin D in participants. The findings suggested that patients with hypothyroidism, autoimmune thyroid disease, and Hashimoto’s thyroiditis had lower vitamin D levels compared to healthy individuals.^28^

 The possible underlying mechanisms could be explained through role of obesity. Weight gain plays a critical role in the development of NAFLD in obesity, primarily by altering the structure and function of AT.^49^ Excessive body weight leads to the accumulation of AT, impaired adipocyte function, the development of adipocyte hypertrophy, and an altered profile of adipokine secretion.^50^ AT is composed of various immune cells that contribute to immune homeostasis.^51^ Adipocytes hypertrophy disrupts blood flow and leads to necrotic cell death, localized hypoxia, and infiltration by inflammatory macrophages.^52^ As a result, enlarged adipocytes produce lower levels of adiponectin and higher levels of monocyte chemotactic protein-1 (MCP-1), Interleukin 1 beta (IL-1β), Interleukin 6 (IL-6), Interleukin 8 (IL-8), and tumor necrosis factor α (TNF-α).^53^ Adipokines (leptin, adiponectine) are produced by adipocytes and play a role in regulating carbohydrate and fat metabolism.^50^ Furthermore, vitamin D in AT reduces gluconeogenesis, increases HDL cholesterol levels, modifies the adipokine profile, and elevates leptin levels by influencing the expression of genes regulating the secretion of leptin and adiponectin.^54^ It affects insulin sensitivity through a number of mechanisms, such as stimulating the expression of insulin sensitivity genes by interacting with the VDR receptor located in the cell nucleus.^55^ Therefore, the transcriptional activity of the insulin receptor gene increases which results in an increase in the total number of insulin receptors without any change in their affinity.^54^ Furthermore, they are key predictors of impaired insulin sensitivity- which reduces gluconeogenesis in the liver and increases glucose transport into muscles- correlate with lower vitamin D levels and an inverse relationship with IR.^56,57^ The release of free fatty acids and pro-inflammatory molecules from stressed fat cells into the bloodstream causes chronic inflammation and abnormal fat deposition in ectopic sites, mainly in the liver.^58^ On the other hand, AT also serves as the main storage site for vitamin D and contains critical enzymes involved in vitamin D metabolism.^55^ An active form of vitamin D (1,25(OH)2D) can enhance insulin sensitivity by activating the peroxisome proliferator-activated receptor delta.^59^ Vitamin D insufficiency results in elevated levels of parathyroid hormone, lipogenesis and fat mass whereas reduced insulin sensitivity.^59^ In addition to its metabolic roles, vitamin D is crucial for immune regulation, and its deficiency has been associated with several autoimmune diseases, including disorders affecting the thyroid gland.^39^ In autoimmune hypothyroidism, low 25(OH)D levels may exacerbate immune dysregulation, potentially triggering or worsening autoimmune thyroid destruction.^31^ Unlike autoimmune thyroid disease, there is no immune-mediated mechanisms in non-autoimmune hypothyroidism, which might explain the lack of a significant association with vitamin D levels in the present study.^31^ Vitamin D exerts its effects by activating VDRs, which not only regulate specific genes but also as found in various tissues (such as thyroid, pancreas, and cardiac tissues). The receptors have role in the function of these organs.^61,62^ The potential connection between thyroid function and vitamin D levels could be probably due to that both vitamin D and THs interact with similar steroid and nuclear hormone receptors and have similar physiological responses.^63^ Therefore, low levels of vitamin D may worsen the systemic abnormalities linked to hypothyroidism.^64^

 A notable strength of this study is its case-control design, which is appropriate for evaluating the association between vitamin D deficiency and non-autoimmune hypothyroidism in patients with NAFLD. The inclusion of overweight/obese female participants and data collection during the fall and winter months, when seasonal variation in vitamin D status is relatively limited, may also be considered methodological strengths. Restricting data collection to this period helped minimize the potential influence of seasonal fluctuations in sun exposure on serum vitamin D concentrations.

 A notable strength of this study is its methodological design- case-control study- which provide a a stronger design for investigating cause-effect link compared with cross-sectional ones. The inclusion of overweight/obese female participants and the collection of data during the fall and winter months, when vitamin D levels are less influenced by seasonal fluctuations in sun exposure, may be considered methodological strengths. Collecting data within this relatively restricted period helped minimize seasonal variation in vitamin D status among participants. Nevertheless, the present study was not a population-based survey; therefore, the generalizability of the findings to the broader population is limited. In addition, only female participants were included in this study. Accordingly, our findings may not be generalizable to males or to normal-weight individuals with NAFLD. Future studies including more diverse populations are warranted to confirm the present findings.

## Conclusion

 In conclusion, no significant association was observed between serum TSH levels and vitamin D deficiency among overweight/obese female patients with NAFLD. Low sun exposure was significantly associated with VDD; however, given the observational case-control design of the present study, no causal relationship can be inferred. Further prospective studies are needed to clarify the temporal relationship between vitamin D status, thyroid function, and sun exposure, as well as to elucidate the underlying biological mechanisms.

## Competing Interests

 The authors declare no conflict of interest.

## Ethical Approval

 The study protocol was approved by the Ethics Committee of the Tabriz University of Medical Science (IR.TBZMED.REC.1402.617). Written informed consent was obtained from all participants prior to enrollment in the study.
